# Soy Lecithin-Derived Liposomal Delivery Systems: Surface Modification and Current Applications

**DOI:** 10.3390/ijms20194706

**Published:** 2019-09-23

**Authors:** Ngoc Thuy Trang Le, Van Du Cao, Thi Nhu Quynh Nguyen, Thi Thu Hong Le, Thach Thao Tran, Thai Thanh Hoang Thi

**Affiliations:** 1 Institute of Research and Development, Duy Tan University, Danang 550000, Vietnam; thuytranglengoc@gmail.com; 2 Faculty of Pharmacy, Lac Hong University, Buu Long Ward, Bien Hoa City, Dong Nai Province 810000, Vietnam; caovandulhu@gmail.com (V.D.C.); ds.nhuquynhnguyen@gmail.com (T.N.Q.N.); Hongle5792@gmail.com (T.T.H.L.); tranthachthao185@gmail.com (T.T.T.); 3 Biomaterials and Nanotechnology Research Group, Faculty of Applied Sciences, Ton Duc Thang University, Ho Chi Minh City 700000, Vietnam

**Keywords:** soy lecithin, liposome, drug delivery system, surface modification

## Abstract

The development of natural phospholipids for nanostructured drug delivery systems has attracted much attention in the past decades. Lecithin that was derived from naturally occurring in soybeans (SL) has introduced some auspicious accomplishments to the drug carrying aspect, like effectual encapsulation, controlled release, and successful delivery of the curative factors to intracellular regions in which they procure these properties from their flexible physicochemical and biophysical properties, such as large aqueous center and biocompatible lipid, self-assembly, tunable properties, and high loading capacity. Despite the almost perfect properties as a drug carrier, liposome is known to be quite quickly eliminated from the body systems. The surface modification of liposomes has been investigated in many studies to overcome this drawback. In this review, we intensively discussed the surface-modified liposomes that enhancing the targeting, cellular uptake, and therapeutic response. Moreover, the recent applications of soy lecithin-derived liposome, focusing on cancer treatment, brain targeting, and vaccinology, are also summarized.

## 1. Introduction

The field of nanotechnology has provided the medical research fraternity with viable systems of delivering therapeutic substances, antioxidants, and nutrients to targeted areas of the body [1,2]. Consequently, a wide range of nanomaterials has been developed and their efficacy and safety as delivery systems tested [3,4,5,6,7,8]. Among different types of carriers, liposome has been discovered and subsequently developed and applied in the medical field [9,10,11]. The special place that liposomes occupy as drug delivery systems is largely attributed to their capacity to encapsulate materials of different natures, polarities, and sizes [12,13,14]. The encapsulation capacity is enhanced by the presence of phospholipid bilayers within their structure. According to the first person that discovered liposomes, that is, Alec Bangham, he described them as swollen systems made up of phospholipids [15]. Liposomes can be easily distinguished from micelles and other lipid phases by using molecular (e.g., fluorescent) spectroscopy methods [16,17]. It is notable that the use of conventional liposomes has been associated with major limitations. The limitations include the inability of conventional liposomes to stay in circulation for a longer time to allow for the slow release and enhanced bioavailability of therapeutic substances [18]. For example, conventional liposomes that are intravenously administered enter the reticuloendothelial system (RES) where they are detected as foreign materials by the serum components of the RES, specifically opsonin [19,20]. Once they are recognized as such, phagocytes within the RES respond by destroying the liposomes. The outcome of this cascade is a short circulation time for conventional liposomes, which affects their therapeutic efficacy of drug encapsulated in liposomes [21]. Similar challenges are encountered when conventional liposomes are administered through the oral route. The gastrointestinal tract presents harsh conditions that limit the availability and stability of the liposomes [22].

Presented with these challenges, it has become a research priority to develop mechanisms of enhancing the stability of liposomes. Furthermore, liposomes are highly preferable as carriers of therapeutic substances due to their low toxicity profiles, flexibility in their encapsulation of a wide range of substances, and high biocompatibility [23,24]. Consequently, significant advances have been made in liposomal design, which have resulted in the availability of improved systems not only for therapeutic, but also for diagnostic, cosmetic, and nutritional applications [25,26,27]. For example, improved liposomal designs for magnetic resonance imaging have been created for the purposes of improving diagnosis at the molecular and cellular levels [28]. Similarly, various studies have confirmed the usefulness of liposomes in the treatment of a variety of diseases when combined with specific drug formulations [21]. The present review contends that researchers have applied specific techniques to achieve surface modification and functionalization of liposomes with different anchoring lipids or antibodies in order to circumvent the limitations associated with conventional liposomes as highlighted above. As such, the review begins by exploring the recent studies that demonstrate the application of surface modification strategies for purposes of enhancing the targeting, uptake and therapeutic response mediated by liposomes. In this respect, PEGylated liposomes, ligand-targeted liposomes, and multifunctional liposomes shall be discussed from the perspective of current studies on liposome surface modification. The second section of the review will focus on soybean lecithin liposome-based carrier systems that have been developed for specific applications. Moreover, the choice of phospholipids from natural sources, such as soybeans and egg yolk, is often influenced by considerations of the liposome properties, as determined by bilayer components. For example, soy lecithin facilitates the large-scale production of liposomes that are much more permeable than egg yolk lecithin [29,30]. The last section of the review will discuss the current applications of liposome surface modification for cancer treatment, brain targeting, and vaccinology.

## 2. Soy Lecithin-Derived Liposome (SLP): From Competitive Advantage to Potential Delivery System

**Figure 1:**
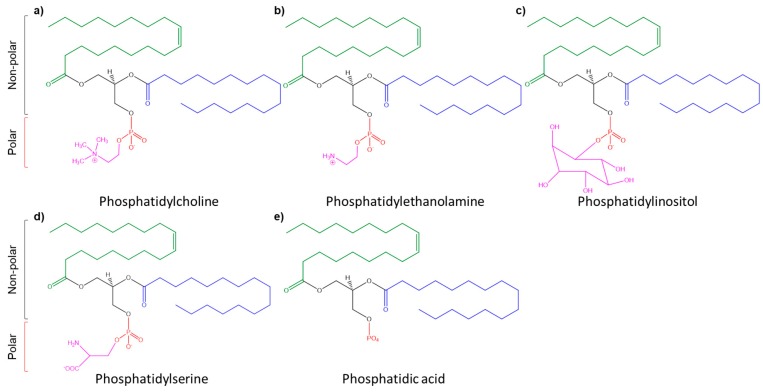
Chemical formulas of different phospholipid-derived lecithin. Shown in red—phosphate group; green—monounsaturated fatty acid; blue—saturated fatty acid; pink—choline (**a**), ethanolamine (**b**), inositol (**c**), serine (**d**), and glycerol (**e**).

Lecithin generally refers to the various triglycerides, phospholipids (phosphatidylinositol, phosphatidylcholine, phosphatidylethanolamine), and glycolipids (Figure 1) [31]. However, the term lecithin, as applied in the field of biochemistry, specifically refers to pure phosphatidylcholine, which is a phospholipid that is derived from the phosphate fraction extracted from vegetables such as soybeans, sunflower, rice beans, and rape seed [32,33]. Natural phospholipids can also be extracted from animal sources, including egg yolk, marine sources, and milk [32]. For liposomal formulation, lecithin from sources other than soybean carries certain drawbacks. For example, lipids that are bovine or egg-derived have stability issues because of their high concentration of polyunsaturated fatty acids [34,35]. Therefore, lecithin from these sources is less stable when compared to soybean lecithin and other plant-derived lecithin, which contain less polyunsaturated fatty acids [32,36]. Additionally, bovine and egg-derived lecithin (e.g., egg-yolk, and milk lecithin) present the possibility of protein contamination and/or pathogenic contamination, such as viruses [34]. In this regard, such lecithin sources might require additional testing to ensure contamination-free liposomes.

On the other hand, lecithin that was extracted from soybeans is considered to be more economical, safer, and stable from a production point of view [30,37]. Apart from soybeans being produced in large scale across the world, the liposomes from non-purified soybean lecithin can be more economically produced because the non-purified components contain significantly higher amounts of phospholipid components when compared to other vegetable sources [32,38]. Furthermore, Yokota, Moraes, and Pinho note that, on average, a kilogram of purified phospholipids from natural sources costs 980 euros while a kilogram of non-purified soybean lecithin costs 196 euros [39]. This implies that soybean lecithin has the advantages of being more stable (less polyunsaturated fatty acids), safer, abundantly available in both purified and non-purified forms, and less costly for both laboratory and pharmaceutical production of liposomes [40].

### 2.1. SLPs for the Delivery of Caffeine

Budai et al. compared the efficiency of liposomes that were generated from both soy and egg lecithins in the delivery of caffeine to the skin for its antioxidant and soothing benefits in one of the various studies that have investigated the viability of soy lecithin liposome-based carrier systems [41]. Previous studies by Mustapha et al. and Flaten et al. have shown that the release of caffeine from gel systems depended both on caffeine concentration and the concentration of the material that formed the gel [42]. Specifically, Mustapha et al. conducted a skin permeation study in which a significant flux of caffeine in the skin sites was noted where the polymer that formed the gel was present in lower concentration. Similarly, the study by Budai et al. aimed at exploring how the amount of phosphatidylcholine in liposomes from soy lecithin (SL) and egg yolk lecithin (EL) affected the surface tension, pH, and viscosity of the liposomes. Additionally, the ability of the two types of lecithin liposomes to entrap caffeine was compared. In terms of the surface tension, it was confirmed that both SL and EL are active surfactants, as their surface tension at a lipid concentration of 15 mg/mL were 39.6 mN/m and 39.5 mN/m, respectively. In terms of the pH, multilamellar vesicles that were prepared from egg lecithin had a lower pH (4.0) than liposomes prepared from soy lecithin (7.2) at a lipid concentration of 10 mg/mL. Since the study involved the application of liposomes in dermatology and cosmetics in which the ideal pH is 5.5, both liposomal formulations were found to be applicable, as they deviated from the ideal pH by only 1.5 and 1.7 respectively. When the researchers took the viscosity measurements, both liposomal samples indicated higher viscosity scores at higher lipid concentrations. As such, the type of lecithin did not affect the viscosity of the liposomes at a specific concentration of phosphatidylcholine. The results on the encapsulation efficiency of the two liposomal formulations indicated differences that were not significant to qualify one formulation as better than the other in the delivery of caffeine to the skin. Moreover, SL had an encapsulation efficiency of 29.2 ± 3.1%, while that of EL was 31.2 ± 4.4% [41]. The results corresponded with those of Memoli et al., who also found similar encapsulation efficiencies for calcein in the two lecithins [43].

### 2.2. SLPs for the Delivery of Methanolic Neem Extract

The use of liposomal formulations from soy lecithin for drug delivery to the skin has been studied further by Singh, Vengurlekar, and Rathod [44]. According to the researchers, soy lecithin liposome-based carrier systems are highly potent as delivery vehicles for herbal drugs, such as Methanolic Neem Extract (MeNE), which is derived from the leaves of *Azadirachta indica* [44,45]. Moreover, alcoholic extracts from the leaves of this herb have been found to possess good anti-microbial properties that can be harnessed for topical use against skin infections. It was important to encapsulate the herbal drug in an efficient liposomal neem gel in order to improve the delivery efficiency and efficacy of MeNE. The liposomal gel was prepared while using soy lecithin, cholesterol, MeNE, and a phosphate buffer. The first step in the formulation of the liposomes was to prepare a lipid phase, which was achieved by the dissolution of accurately weighted amounts of soy lecithin, cholesterol (4:1), and MeNE (80 mg) in a mixture of chloroform and methanol (ratio by volume; 2:1 v/v) in a 250 mL flask with glass beads. A rotary evaporator was then used to generate a dry thin film of lipids on the surface of the glass beads and walls of the glass flask, which was then hydrated at 60 degrees Celsius while using Phosphate buffer of pH 6.5. The dispersion that formed was then left to settle for about 3 h to facilitate maximum swelling of the film so as to obtain vesicular suspension of lipids. Physicochemical evaluation of the liposomal gel indicated particle size of approximately 3.2 micrometers and a pH of 6.5. Additionally, the soy lecithin liposome-based delivery system was found to entrap MeNE at an efficiency rate of 69.52% and drug diffusion rate of about 62.2% after 24 h. At the same time, the liposomal delivery system was able to induce a 20% skin retention rate of MeNE after 24 h. It is notable that the deposition of other liposomal components apart from the drug into the skin has the ability to increase the skin’s capacity to retain the drug. While using a rat model, the liposomal gel did not show any signs of irritation on the skin. In terms of its stability (drug release, flow, and physical appearance), the soybean lecithin liposome-based MeNE delivery system remained stable for three months under storage temperature of between 2 and 8 degrees Celsius [44]. These results confirmed a constraint hydrolysis rate of lipid by cold temperature. ′Similar trends were also observed for the case of water-soluble compounds from *C. sinensis* CS1197 loaded nano-liposomes, liposomes stored at 4 and 25 °C showed stable after eight weeks [46]. Muppidi et al. study showed the same result when developed liposomal vancomycin formulations. The conventional and PEGylated liposomal formulations were both stable at 4 °C for three months [47]. This is true for lipid phase transitions, lipid bilayers exist, such as DPPC in the gel phase (Lβ’) at temperatures below 35 °C, whereas above 42 °C, they are present in the liquid crystalline phase (Lα’). Between 35 and 42 °C, the phospholipid bilayer is in the Pβ’ or so-called “rippled phase”. The pretransition corresponds to a reorganization of individual lipid molecules in the lipid bilayer. Following the pretransition at 35 °C, several conformational changes occur in the lipid molecules as well as changes in the geometry of the lipid bilayers leading to the liposome lost its stability. Therefore, in this study, further research needs to be done by various techniques, such as DSC, FT-IR, and NMR, in order to study the thermal effects of additives in bilayer membranes and neem’s bioactive compounds-biomembrane interactions.

### 2.3. SLPs for the Delivery of Antimalarials

Soy lecithin liposome-based carrier systems have also been developed for the delivery of antimalarial agents as described in one of the recent studies by Rajendran et al. [48]. The researchers acknowledge the fact that combating malaria at the global level has been difficult due to the development of resistance against antimalarial drugs. Liposomal formulations from soy lecithin provide a potent mechanism of encapsulating important drugs, such as monensin and delivering them into the body in a way that circumvents drug resistance by the malaria parasite, *Plasmodium falciparum,* in order to make the fight against malaria more effective. The study by Rajendran et al. aimed to evaluate the antimalarial activity of monensin in the context of its encapsulation in soy lecithin liposome-based drug delivery system. Therefore, the lipid formulations of phosphatidylcholine (PC) from soybean in combination with cholesterol containing either phosphatidic acid or stearyl amine were prepared together with varying densities of DSPE-mPEG-2000 (Rajendran et al.). After the incorporation of monensin into the liposomes, in vitro determination of parasite killing capacity of monensin was achieved by using hypoxanthine incorporation assay. Additionally, in vivo evaluation of parasite killing was achieved using infected red blood cells that were Giemsa-stained. In terms of the physicochemical properties of the soy lecithin liposomes, the sizes of their spherical shapes ranged from 90 nm to 120 nm based on high-resolution electron microscopy and dynamic light scattering. The results of the antimalarial activity showed a heightened parasite killing capacity of monensin in liposomes containing DSPE-mPEG-2000 (5 mol%), stearyl amine, and free artemisinin. Furthermore, this combination resulted in improved survival of infected erythrocytes and the prevention of parasite recrudescence [48]. It is worth noting that, apart from the occurrence of drug resistance, efforts to eradicate malaria have been hampered by the poor solubility of most antimalarials in plasma, low accumulation in erythrocytes, and large biodistribution volumes [49]. As a result, the drugs’ ability to fight plasmodia in erythrocytes has been limited. Therefore, the study by Rajendran et al. provides an avenue of further exploring the usefulness of soybean lecithin liposome-based formulations in the delivery of antimalarial drugs. Moreover, the study showed superior anti-plasmodial activity, preferential drug uptake by infected cells, and the absence of hemolysis of red blood cells upon encapsulating monensin in soya PC: Chol-liposome [48].

### 2.4. SLPs for the Delivery of Anti-TB Drugs

Soy lecithin liposomes, as drug carriers, have been additionally studied in the treatment of tuberculosis (TB). According to the 2017 global report on tuberculosis by the World Health Organization, out of the 9 million new cases of TB reported in 2016, approximately 500,000 were attributed to multidrug-resistant (MDR) strains of *Mycobacterium tuberculosis* (WHO). Therefore, current research in the fight against TB is centered on devising means of formulating new antimicrobials and more efficient mechanisms of delivering the drugs at high concentration inside the macrophages of the lungs where TB bacteria are active [50]. According to Islan et al., nanodevices, such as liposomes, provide the much needed biological mechanism of achieving targeted administration of anti-TB drugs at lower dosages and with minimal side effects while circumventing the drug resistance mechanisms of *M. tuberculosis* strains [51]. Inhaled drugs are preferable as therapeutic strategies, because they are able to reach the cavitary lesions of the bronchial tree where bacteria are overtly present and where strains of *M. tuberculosis* rapidly multiply [50]. With the help of liposomes, the half-life and targeting efficiency of anti-TB therapies can be enhanced when compared to inhalable dry powder formulations with no liposomes [52]. However, previous studies have recorded certain difficulties in anti-TB drug entrapment in liposomes. For example, in a study that involved the passive encapsulation of pyrazinamide and isoniazid at the stage of lipid film hydration, Justo and Moraes were able to obtain a drug entrapment efficiency of only 2.2% for pyrazinamide and 2.5% for isoniazid [53]. When ethionamide was used for incorporation in the lipid film, the trapping efficiency increased to 42%, but the equivalent molar ratio of drug: lipid of 0.04 was too low to achieve the expected therapeutic benefits [53].

Despite the challenges of the aforementioned studies, more recent studies have shown the inherent potential of soy lecithin liposomes for the efficient encapsulation and deeper deposition of anti-TB aerosol therapy. One of these studies by Zaru et al. involved the use of liposomes that were formulated from soy lecithin to entrap rifampicin for the passive targeting of alveolar macrophages that were infected by Mycobacterium avium complex (MAC) [54]. Using rifampicin-loaded soy lecithin liposomes that had been freeze-dried, as well as a mixture of Phospholipon 90 (commercially available soy phosphatidylcholine) containing or lacking cholesterol, Zaru et al. reported the stable loading of rifampicin in soy lecithin liposomes only when using liposomes without cholesterol. However, Phospholipon 90 (P90) vesicles were able to encapsulate rifampicin effectively when the liposomal formulations contained P90 and cholesterol in the molar ratios 4:1 or 7:1. The researchers evaluated the nebulization stability and drug retention efficiency of the liposomal formulations after generating rifampicin-liposome aerosols while using a compressor-driven nebulizer. All of the formulations had good stability and a 65% retention rate of rifampicin. Additionally, rat models showed that aerosol inhalation of the liposome-encapsulated drug was able to reach the lower airways and acted against Mycobacterium avium complex by inhibiting the growth of the pathogens within the lung macrophages [54].

Research has demonstrated the need to overcome the challenge of loss of stability in the encapsulated drugs during the development of soy lecithin liposome-based drug delivery systems. In a bid to address this issue, some researchers have tested the role of pro-liposomes (a free-flowing combination of granular products comprising phospholipid precursors and the drug of interest) in promoting the stability of the entrapped drugs. Moreover, pro-liposomes are convenient to use, as they easily convert into highly stable liposomes upon hydration and subsequent reconstitution [55]. One of the methods that is used to prepare pro-liposomes is referred to as Quality by Design (QbD) [56]. In this systematic approach, drug products are developed and the final product is achieved through a well-defined evaluation of parameters [56].

Patil-Gadhe and Pokharkar reported a study that demonstrated the application of soy lecithin in the preparation of pro-liposomes using the QbD approach [57]. The QbD principles were applied in the study to evaluate the performance of proliposomes formulated using soya phosphatidylcholine (SPC) that had been hydrogenated and loaded with rifapentine (RPT). The goal of the study was to optimize the delivery of this old drug in the treatment of TB through pulmonary inhalation. The method of spray drying was used in a single step fashion to prepare the RPT-loaded proliposomes before optimizing the independent variables by the factorial design method. While using multiple regression and contour plots, the dependent variables (fine particle fraction (FPF), size of liposome vesicle, mass median diameter, and mass median aerodynamic diameter (MMAD)) were studied in terms of how they were affected by the independent variables (soya phosphatidylcholine (hydrogenated), charged lipid (stearyl amine), and drug (RPT)). The optimized formulation that gave rise to respirable proliposomes comprised rifapentine-loaded proliposomal dry powder for inhalation (R-LDPI-7): SPC (hydrogenated) in the ratio 1:2, as well as stearyl amine. The resultant proliposomes had a fine particle fraction of approximately 92.5% and MMAD of 1.56 micrometers. The pharmacokinetics of R-LDPI-7 following pulmonary inhalation demonstrated sustained release of rifapentine and a seven-fold drug retention time in the lungs compared to dry powder combined with rifapentine without the use of proliposomes (R-DPI-0) [57].

### 2.5. SLPs for the Delivery of Metronidazole

Metronidazole is an important antiprotozoal and antibacterial agent that is often prescribed to treat bacterial vaginosis [58]. Moreover, metronidazole that is locally administered is beneficial, because it circumvents the side effects associated with the systemic administration of many antibacterials [59]. In most cases, metronidazole for intravaginal use is available as a gel that is the equivalent in concentration to the synthetic form of the drug [58]. The efficacy of the antibacterial agent is challenged by the fact that its stay at the application site is relatively short despite the availability of metronidazole for local administration. For antimicrobial agents to have the desired therapeutic impacts, their residence time at the infected sites should be prolonged [60]. For this reason, bacterial vaginosis can be more effectively treated by developing systems of delivering antibacterials in a way that prolongs their contact with the mucosal surface of the vagina. At the same time, such systems should facilitate a sustained release of the drug that has been incorporated. In this respect, liposomes that were formulated using soybean lecithin have been developed for the delivery of metronidazole.

In one of the studies, Patel and Patel formulated liposomes while using a combination of cholesterol and soy lecithin for purposes of encapsulating metronidazole and testing the drug’s stability and the efficiency of drug release from the liposomes [59]. The liposomes were formulated by dissolving carefully measured amounts of cholesterol and soy lecithin in a flask containing 8 mL solution of methanol and chloroform (equivolume ratio of 1:1) at approximately 60 degrees Celsius. In a separate set up, a metronidazole drug solution was prepared in 2 mL of methanol and then added to the 250 mL flask containing cholesterol and soy lecithin solution. The resultant mixture was then rotated for ten minutes at 100 revolutions per minute. The resultant thin film of dry lipid was converted into a suspension that would be hydrated to form lipids. 1% of carbopol 934P bioadhesive gel was used to incorporate the liposomes in order to improve liposomal stability and application viscosity. This was followed by an evaluation of how the drug entrapment and release was affected. In terms of the sizes of the liposome vesicles, the average size ranged between 11.77 and 15.71 micrometers. Additionally, the majority of the liposomes containing metronidazole had a spherical shape. Analysis of drug entrapment revealed a maximum entrapment of 36.45%. The low efficiency in the encapsulation of metronidazole can be attributed to the low solubility of the drug in both lipophilic and hyrophilic media. Despite the relatively low efficiency in the entrapment of metronidazole, the soybean lecithin liposomes showed a biphasic pattern of drug release. In the biphasic pattern, there was an initial phase of rapid release, followed shortly by a continuous/prolonged phase of sustained release [59].

### 2.6. SLPs for the Delivery of Casein Hydrolysate

Protein hydrolysates are valuable products for their nutritional applications, as they can be combined with various food formulations to enhance the nutritional value of food. Furthermore, certain protein hydrolysates can be used to improve the biological and physiological functions in the body in addition to reducing the risk of various diseases [39]. It is also worth noting that certain bioactive peptides in the form of protein hydrolysates are recommended as additives to food formulations meant to support the specific needs of patients with clinical diagnosis of diseases, such as phenylketonuria, ulcerative colitis, and chronic liver disease [61]. Among the clinical benefits of protein hydrolysates include antioxidant activities, lowering of cholesterol levels, and improvement in mineral absorption [62]. Despite the proven benefits of protein hydrolysates such as casein hydrolysate, certain challenges that are related to their consumption suitability and incorporation in food substances exist. For example, the sensory properties of these bioactive peptides are unpleasant in that some of them have strong odor and bitterness [39]. In addition, it is difficult to achieve a continuous distribution of their particles in aqueous media due to their hydrophobic properties. Another challenge is the allergenicity of the protein derivatives. Oral administration of the bioactive peptides is further challenged by reduced bioavailability due to their poor intestinal permeability and instability in the gut [39].

Yokota et al. reported an experiment in which the peptide was microencapsulated in liposomes formulated using crude soy-lecithin in order to overcome these obstacles and ensure convenient and effective delivery of casein hydrolysate (CH) [39]. The aim of the study was to determine the feasibility of using non-purified soy lecithin (Lipoid S40) to generate liposomes for the laboratory application in casein hydrolysate encapsulation. The liposomes were prepared by the method of film hydration, in which the hydration step was accomplished while using casein hydrolysate solubilized in a buffer. Trehalose or sucrose was then added to the resultant fluid to attain a mass ratio of phospholipid: cryoprotectant; 1:4. On evaluating the vesicle size and encapsulation efficiency, the researchers noted that liposomes that were formulated using crude soy lecithin and non-cryoprotected had smaller sizes compared to liposomes formulated using purified soy lecithin (Lipoid S100-H) (Table 1). The researchers concluded that phosphatidylcholine present in crude soy lecithin (12–15 percent by mass) caused the packing of phospholipids to be tighter, thus contributing to smaller particle size. In terms of the encapsulation capacity, non-cryoprotected liposomes (both Lipoid S40 and Lipoid S100-H) showed a higher efficiency than cryoprotected liposomes. However, the higher encapsulation capacity of the non-cryoprotected liposomes would be eroded by their instability once introduced into the biological systems of the body [39]. Therefore, the idea was to demonstrate that lyophilized liposomes formulated from crude soy lecithin would carry the advantage of being more stable, despite lower encapsulation efficiency.

Some of the common problems that arise with liposomes are entrapment inefficiency and drug release with the required profile. However, literature has identified several strategies through which the issues can be solved. One method is loading liposomes with isoniazid. According to a study by Roy et al., liposomes of isoniazid, which are made by thin-layer film hydration, showed entrapment efficiency, which was determined by the particle sizes viewed from scanning electron microscopy (SEM) photographs [63]. The process involved the use of isoniazid or soya lecithin (L-a-phosphatidylcholine) and chloroform. The results proved that the isoniazid liposomes had reduced oxidation and hydrolysis, which are the main causes of chemical instability. Furthermore, when stored at low temperatures and light preservation, liposomes can avoid the reactions. Moreover, isoniazid liposomes were associated with proper entrapment and stability due to pH adjustments, temperature, ionic interaction, and application of cholesterol in the bilayer structure [63]. Therefore, loading liposomes with isoniazid can be used to solve entrapment issues with liposomes. Moreover, suing X-ray-triggered Liposomes, together with gold nanoparticles and photosensitizer verteporfin, can solve drug release problems. Co-embedding photosensitizers and nanoparticles (3 to 5 nm) inside a lipid bilayer design the X-ray-triggered liposomes [64]. Notably, gold is significant in the process due to its high atomic number and strong interaction with X-ray radiation. Photosensitizers are also a source of reactive oxygen species (ROS), which oxidize unsaturated lipids and destabilize liposomal membranes. The procedure is monitored through highly specific fluorescence probe to detect oxygen generation. The process improves drug release by oxygenating unsaturated lipids, resulting in the disruption of the Liposome structure [64]. The strategies offer ways through which entrapment inefficiency and drug release issues that are associated with Liposomes can be solved.

The discussion on the use of soybean lecithin liposome-based drug delivery systems shows huge potential in the treatment of a wide range of diseases that affect the human population. From skin infections to metabolic disorders, such as phenylketonuria, the relevance of soy lecithin liposomes in the encapsulation and delivery of specific molecules has been proven.

## 3. Surface-Modification Strategies

**Figure 2:**
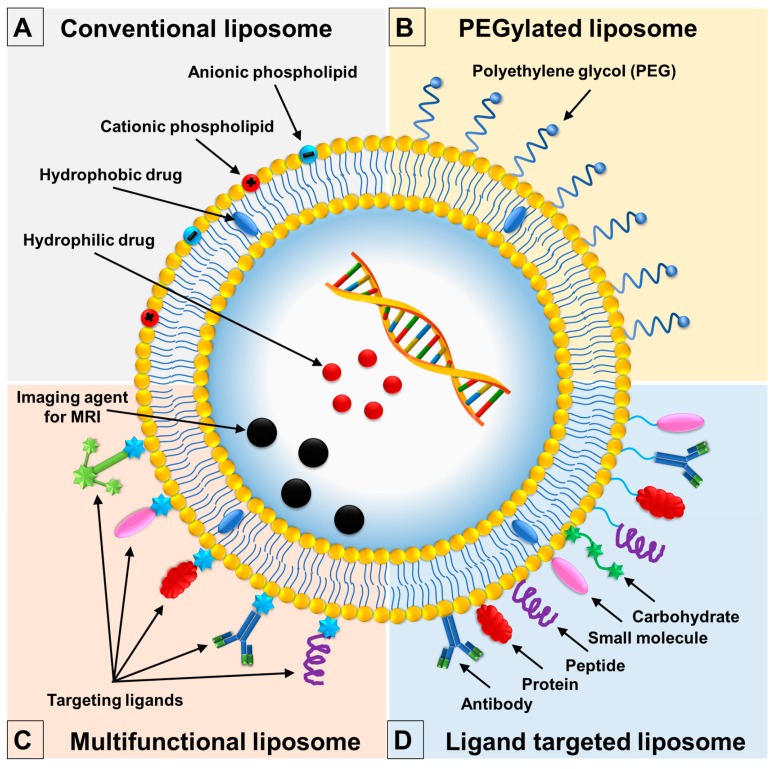
Classification of liposomes and the surface modification strategies applied in each category. (**A**) Conventional liposomes simply contain: neutral, anionic, and cationic phospholipids; (**B**) stealth liposomes are PEGylated contain a polyethylene glycol (PEG) layer; (**C**) multifunctional liposomes have modified surfaces in addition to carrying imaging agent for diagnostic purposes (diagnosis and treatment functions); and, (**D**) targeted liposomes have modified surfaces through the attachment of targeting ligands (antibody, protein, peptide, small molecule, carbohydrate). [21].

One of the limitations of conventional liposomes is their short time of circulation in the blood, as they are easily detected as foreign materials and degraded by components of the immune system (Figure 2A). Consequently, the degraded liposomes accumulate in vital organs [65,66]. The accessibility of passively targeted liposomes to these tissues is as follows: spleen and lymaphatics > endocrine tissues > nonendocrine organs [67]. A previous study indicated that liposomes with a size range of 100–200 nm exhibited a 4-fold higher rate uptake in the tumor as compared to liposomes of <50 nm or >300 nm [68]. The lower accumulation of small liposomes (<20–30 nm) might be explained by their high permeability. Small size of liposomes allows for them not only to rapidly pass through the capillary wall, but also be returned to the blood circulation by diffusion, in other words. In contrast, the lower uptake of larger liposomes can be attributed to the size-limited permeability [69,70,71,72,73,74]. Over the years, researchers in liposomal drug delivery have discovered that by altering their surface functionalization, liposomes can become more effective and efficient vehicles of delivering useful therapeutic substances in a targeted way [75,76]. Furthermore, surface modification strategies of liposomes, such as PEGylated liposomes, ligand-targeted liposomes, and multifunctional liposomes, have proven to be a reliable way of overcoming the challenges that are associated with conventional liposomes [77].

### 3.1. PEGylated Liposomes

**Figure 3:**
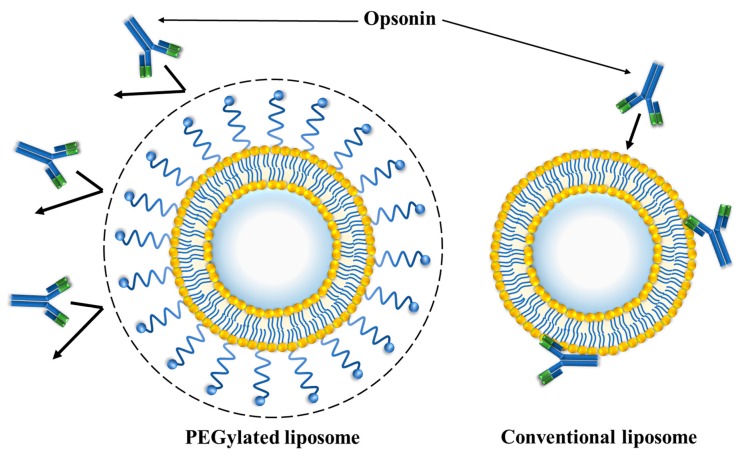
Comparison of PEGylated liposome with conventional liposome. PEGylated liposome: high molecular size, high solubility, and shielding against the recognition by opsonin. Conventional liposome: small molecular size, low solubility, and recognition by opsonin.

As a way of managing this limitation, various researchers have investigated the effectiveness of liposomes that are coated with polyethylene glycol (PEG), which is a hydrophilic polymer (Figure 2B). The role of PEG coating on liposomes is to increase the repulsive forces that are created between serum components and the liposomes [78,79]. As a result, liposomes that are PEGylated are also referred to as stealth liposomes, because they become extremely difficult to detect by phagocytes and other components of the immune system (Figure 3) [21]. The outcome of their stealth nature is that they accumulate in the targeted sites, thus promoting their efficacy. Moreover, PEGylated liposomes have lower aggregation rates in the bloodstream and a high degree of storage, which increase bioavailability of liposomes [80,81]. Additionally, in recent research, charged lipid components were noticed that affect the particle size of liposome. Stella et al. exploited such characteristics in a 2018 paper when they prepared conventional and PEGylated shikonin-loaded liposomal formulations [70]. The use of DSPE-mPEG_2000_ resulted in PEGylated formulations had smaller particle sizes and lower polydispersity indexes to conventional samples where a similar finding was also noted previous studies [71], confirming the superiority of PEGylated formulations over conventional ones. An observed decrease in particle size was explained by the increasing of PEG in the lipid bilayer, causing a reduction in the intensity of lateral repulsion of bilayer and an increase in interlamellar repulsion. Many studies with other bioactive compounds have confirmed this trend [72,73,74].

One of the studies that have tested the effectiveness of PEGylated liposomes is by Biswas et al. [82]. In this study, liposomes that had been modified by stearyl triphenylphosphonium (STPP) were reported to exhibit non-specific cytotoxicity. A conjugate comprising of polyethylene glycol and phosphatidylethanolamine (PEG-PE) was synthesized in order to overcome this problem and ensure that STPP-modified liposomes only targeted the mitochondria. The triphenylphosphonium (TPP) component of STPP was then attached to the PEG-PE conjugate at the distal end to form TPP-PEG-PE block. The resultant conjugate was then inserted into the lipid bilayer of the liposome to form modified liposomes for the delivery of paclitaxel (PTX) through the specific targeting of the mitochondria [82]. Additionally, the researchers used in vitro and in vivo tests to evaluate the efficacy of drug delivery and the toxicity of the modified liposomes. It was noted that liposomes whose surfaces had been grafted with 8 mol% of TPP-PEG-PE exhibited less cytotoxicity compared to liposomes whose surfaces had been modified with STPP only. Additionally, the presence of the PEG-PE block in the conjugate further lowered the non-specific cytotoxicity that was initially seen in STPP-modified liposomes as compared to when only PEG and STPP were used to modify the liposomes. Nevertheless, the incorporation of PEG in TPP to create TPP-PEG liposomes achieved efficient targeting of mitochondria with the cancer drug, as confirmed through confocal microscopy that used stained mitochondria. Further, the surface modification of liposomes using the TPP-PEG conjugate was sufficient for achieving high anti-tumor efficacy in vitro as compared to unmodified liposomes loaded with PTX [82].

### 3.2. Ligand Targeted Liposomes

In addition to surface modification of liposomes using PEG, liposomes have been modified and their functionalization enhanced through engineering techniques that facilitate the attachment of a targeting ligand such as antibodies, small molecules or peptides to the liposomes surface. The attached ligand is able to track the specific receptors that are overexpressed on the surfaces of the diseased cells. By attaching to the surface receptors, the ligands mediate the action of the drug that has been incorporated in the liposome [83,84,85,86]. Such targeted liposomes can be created by directly attaching the targeting ligand on the liposomes surface or on the distal end of polyethylene glycol chains, as illustrated in Figure 2D.

Among the advantages of modifying the surfaces of liposomes by using targeted ligands is that such liposomes are highly specific in terms of targeting the diseased cells. As a result, healthy tissues are spared from the off-target effects that are associated with certain therapies, such as chemotherapy in cancer treatment [87]. Immunoliposomes are good examples of targeted liposomes that are formulated by coupling antibodies to the surface of liposomes through chemical means in order to target antigens with a high degree of specificity [88].

In 2010, Guan’s research developed targeted liposomes by attaching two peptide ligands (^L^CDX and ^D^CDX) with polyethylene glycol molecules on the surface of liposomes for its possibility of biomedical applications in drug delivery systems for the brain [89]. The choice of the two retro-inverso peptides was also based on their higher stability when compared to natural peptides. Moreover, they are able to resist proteolysis in the tissue microenvironment and in blood circulation thus achieving higher targeting efficiency [90]. The application of ^L^CDX and its analog peptide ^D^CDX in brain drug delivery is further supported by their ability to specifically bind to nicotinic acetylcholine receptors of lysosomes of brain capillary endothelial cells, which act as an enzymatic barrier for the transport of peptide ligands to the brain [91]. All of the plain liposomes and liposomes modified with ^L^CDX and ^D^CDX peptide were prepared while using thin-film hydration method. The immunogenicity of the respective liposomes was then evaluated by intraperitoneal injection of BALB/c mice with four weekly doses of the liposomes. The ELISA method was then used to determine the antibodies present in the blood samples while using the antigens ^L^CDX-PEG_3400_-DSPE, ^D^CDX-PEG_3400_-DSPE, or mPEG_2000_-DSPE. The outcomes of this analysis showed that liposomal surface modification with ^D^CDX peptide elicited the strongest immune response as compared to the other liposomal formulations. Precisely, the amount of IgGs generated by ^D^CDX-modified liposomes was 50 times higher than those that were generated by unmodified liposomes and 100 times higher than those generated by ^L^CDX-modified liposomes. The researchers also noted that, after the fourth dose, much more IgMs against the polyethylene glycol coating on the liposome surfaces were produced in ^D^CDX-modified liposomes than in non-peptide/plain and ^L^CDX-modified liposomes [89]. This study illustrated the importance of liposomes modified with peptide ligand by their ability to specifically bind to nicotinic acetylcholine receptor-mediated transcytosis and paves the way for developing a brain-targeted drug delivery system.

In a similar study that involved the modification of liposome surface while using a ligand, Biswas et al. prepared a synthetic polymer from the mitochondriotropic dye rhodamine-123 (Rh123) before joining it to PEG-DOPE conjugate to form Rh123-PEG-DOPE [82]. The use of Rh123 in the modification of liposomal surface was important, because the dye has a high affinity for the mitochondria. In other words, the researchers saw Rh123 as a potentially efficient ligand for targeting the mitochondria within a liposomal system of drug delivery. Moreover, the mitochondrion is a vital organ that controls important physiological processes, such as cellular energy production, cell death, and signaling. As such, a dysfunction in the mitochondrial processes can result in major human diseases, including cancer, diabetes, and neurodegenerative disorders [92]. During the conjugation of Rh123 to the PEG-PE (polyethylene glycol-phosphatidylethanolamine), the PE moiety provided an efficient means of incorporating the resultant polymer (Rh123-PEG-PE) into the membrane of the liposome. At the same time, the PEG moiety acted as the spacer arm for higher flexibility of Rh123, as it interacted with the mitochondria [82]. During the study, liposomes to be used as drug carriers were successfully prepared using lipid film hydration [82]. After the formation of Rh123-PEG_3400_-DOPE, the ampiphilic copolymer was introduced into the lipid bilayer of the liposomes. Experiments that were cell-based could achieve a targeting effect by modifying the liposomal surface with a Rh123 concentration of 1 mol%. Subsequent analysis aimed at establishing the efficiency of liposomal uptake by B16F10 and HeLa cells. The results showed that liposomes whose surfaces had been modified by Rh123-PEG-PE polymer had a higher rate of uptake by both B16F10 and HeLa cells, as determined by FACS analysis and fluorescence microscopy. Similarly, the use of stained mitochondria in the co-localization study showed that modified liposomes accumulated at a higher degree in the mitochondria when compared to plain liposomes [82].

Wicki et al. investigated the effectiveness of ligand targeted liposomes in the form of immunoliposomes (ILs) in disrupting the signaling axis between VEGF and VEGFR2 in tumor angiogenesis [93]. In other words, the researchers aimed to analyze how surface modification of liposomes through monoclonal antibodies against VEGFR2 would affect the efficiency of doxorubicin delivery into the affected cells and tissues. Therefore, the idea was to covalently link anti-VEGFR2 monoclonal antibodies to liposomes that had been modified through polyethyleneglycol (PEG) and that contained doxorubicin: a drug used to treat multiple cancers including colon and breast cancer. Fragment antigen-binding (Fab’) were conjugated by covalent bonding with groups of maleimide compound (H_2_C_2_(CO)_2_NH at the chain termini of PEG-DSPE in order to develop immunoliposomes. The conjugates of monoclonal antibody fragments (Fab’-Mal-PEG-DSPE) were then coincubated with doxorubicin-containing liposomes for half an hour at 55 degrees Celcius. Free drug and conjugates that had not been incorporated were separated from immunoliposomes by Sepharose CL-4B gel filtration. SDS-PAGE was then applied to estimate the efficiency of the incorporation of monoclobal antibody fragments. Using three mouse models (MMTV-PyMT, HT-29, and Rip1Tag2), Wicki et al. tested the effect of the surface modification of liposomes with anti-VEGFR2 antibodies. The researchers noted a superior therapeutic efficacy in immunoliposomes whose surfaces had been modified by the incorporation of anti-VEGFR2 monoclonal antibodies when compared to pegylated liposomes containing doxorubicin, antibodies alone, empty liposomes, and anti-VEGFR2 immunoliposomes with no doxorubicin [93].

Ligand targeted liposomes are created to facilitate the precise delivery of therapeutic agents to diseased tissues, which are tested through imaging techniques. One form of liposomes that is ligand targeted is CDX-modified stealth Liposomes, which are immunogenic. The liposomes are strengthened through the use of peptidomimetics, such as retro-inverso analogs used in targeted drug delivery. In the peptides or all-D retro, the amino acid is retained while the direction of the peptide bonds is reversed. Assembling D-amino acid residues in the reverse order in accordance with the original sequence completes the process. Notably, retro-inverso peptide analogs are stronger and stable as compared to the natural peptides. In brain-targeted drug delivery, CDX-modified liposomes, which are prepared while using the thin-film hydration method and their retro-inverso peptide analog CDX, possess a brain-targeting property by nicotinic acetylcholine receptors that makes them resistant to proteolysis [89]. The methods are tested through several imaging techniques, such as light, polarization, fluorescence, and confocal microscopy. Other ways of testing are electron microscopy techniques, negative scanning, the freeze-fracture technique, cryogenic TEM, and atomic force microscopy (AFM) [94]. The imaging approaches are used to evaluate the morphology of liposomes. Light microscopy provides general details on the shape and size of larger vesicles and the homogeneity of a sample faster than other methods. For instance, TEM is the commonly used technique for examining the morphology of liposomes in detail due to its ability to highlight structural changes [94]. The technique, together with AFM, can image ligands that are conjugated to the surface of liposomes and provide complementary information on surface modifications.

### 3.3. Multifunctional Liposomes

Multifunctional liposomes have been developed and extensively studied for their ability to perform multiple functions through surface modification techniques (Figure 2C). Moreover, the need to diagnose one disease while at the same time treating another cannot be met with liposomal formulations that have single functionality [95,96,97]. In other circumstances, there may be need to administer two anticancer agents: a requirement that single functionality liposomes are not able to satisfy. Therefore, researchers have applied a combination of techniques in surface modification and functionalization to formulate liposomes with a wide range of functionalities. For example, Yuan et al. have reported liposomes that can carry two ligands, such as peptides, in order to perform dual functions [98]. Similarly, Zhang et al. described the application of liposomes with the capacity to carry two anticancer drugs and two ligands, while Erdogan and Torchilin reported the usefulness of liposomes that contain an imaging agent and a targeting ligand [99].

Shihong et al. designed and developed a drug delivery system of theranostic liposomes with multifunctional capacity [100]. By incorporating nuclear imaging, near infrared, and magnetic resonance in the drug delivery liposomal system, the liposomes were designed to achieve diagnosis, monitoring of therapy, and prediction of the treatment outcomes. The study utilized pre-manufactured liposomes that were made up of DSPC, cholesterol, Gd-DOTA-DSPE, and DOTA-DSPE in the ratio 39:35:25:1. A pH and ammonium gradient were applied so that the exterior portion of the liposomes had a medium pH of 7.4, while the interior had a lower pH. The use of a pH/ammonium gradient was important in the modification of the liposomes, because it facilitated the post-loading of imaging radionuclides, such as ^99m^Tc, as well as chemotherapeutic agents (doxorubicin), and therapeutic radionuclide (^186^Re/^188^Re). Additionally, the lipid formulation included DOTA-DSPE for the purposes of enabling the radionuclide labeling within the liposome membrane while using ^64^Cu and β^+^ decay for positron emission tomography imaging. The incorporation of Gd-DOTA-DSPE and DOTA ligand would impart the magnetic resonance imaging capability of the liposomes. The pre-manufactured liposomes were also modified by the insertion of a near-infrared lipidized fluorescent tracer (IRDye-DSPE), which would facilitate the mapping of near-infrared imaging of lymph nodes that are associated with tumors or the tumors [100]. Following the modification of the multifunctional liposomes, the anticancer drug doxorubicin was subsequently post-loaded into the liposomes. Intratumoral injection of the modified multifunctional liposomes in immunodeficient rat models was then accomplished and the resultant magnetic resonance images enabled the visualization of the liposomes in terms of their distribution. Additionally, single photon emission computed tomography radiolabeled with ^99m^Tc, positron emission tomography radiolabeled with ^64^Cu, and near-infrared fluorescent enabled the researchers to visualize the retention and/or distribution of the theranostic liposomes within the tumors.

Yang et al. reported another study that focused on the practical application of multifunctional liposomes [101]. The researchers focused on harnessing the huge potential of small interfering RNA (siRNA) as molecules that can be delivered through modified liposomal systems to disrupt the molecular basis of certain diseases including cancer. The study’s background highlights the fact that the conjugation of siRNA with cell-penetrating peptides (CPPs) to form siRNA-CPPs through disulfide bonds is not effective as a therapeutic strategy. Among the challenges that are faced by siRNA-CPPs include the degradation of CPPs, non-cell specificity and unwanted reduction of the conjugate due to the temperature and redox environment of the cells [101]. Yang et al. developed a liposomal system with the capacity to respond to the dual stimuli (intracellular redox conditions and hyperthermia) encountered by the siRNA-CPP conjugate to overcome these limitations [101]. The liposomes were modified to become thermosensitive by coupling them with a peptide comprising Asparagine-Glycine-Arginine (NGR). The attachment of NGR onto the liposomes was accomplished by combining a mixture of MSPC:DPPC:(DSPE-PEG2000-NGR or DSPE-PEG2000) (weight ratio; 3:87:10) with chloroform followed by evaporation to obtain a lipid film. The lipid film was then hydrated while using HEPES buffer. The conjugate of interest, that is, CPP-siRNA, was formulated by linking CPP to siRNA through disulfide bonds. The conjugate was then incorporated in the thermosensitive liposomes (TSL) containing the NGR peptide. The idea was to “cage” the siRNA-CPPs through encapsulation in the NGR-modified thermosensitive liposomes. By so doing, the siRNA-CPPs would escape premature degradation and reduction before reaching the targeted sites. The results of this study showed that the c-myc genes (the genes that encode transcription factors and regulatory mechanisms in cancer cells) in human fibrosarcoma-cells were silenced by both siRNA-CPPs/TSL-NGR and free siRNA-CPPs *in vitro*. However, siRNA-CPPs/TSL-NGR exhibited superior antitumor efficacy (three-fold) and in silencing c-myc (two-fold) in vivo in xenograft mouse model as compared with free CPP-siRNA under conditions of hyperthermia [101].

Several research groups developed multifunctional as a strategy to transport drugs across the blood-brain barrier (BBB) for treating brain glioma. In 2016, for instance, Xue-tao and co-workers designed, synthesized, and characterized multifunctional targeting vinorelbine plus tetrandrine liposomes that were modified with polyethylenimine (PEI) and vapreotide (VAP) for transporting drugs across the BBB and targeting all glioma cells [102]. In this system, the newly synthesized cholesterol polyethylene glycol polyethylenimine (CHOL-PEG_2000_-PEI) conjugate was modified on the surface of the liposomes and D-a-tocopheryl polyethylene glycol 1000 succinate vapreotide (TPGS_1000_-VAP) conjugate was incorporated onto drugs-loaded liposomes that were used as a targeting molecule for transporting drugs across the BBB and a functional molecule for targeting all glioma cells via receptor-mediated endocytosis, respectively. Besides, tetrandrine was incorporated into the lipid bilayer for inhibiting the multidrug resistance via blocking the expression of P-gp protein that was overexpressed on BBB and glioma stem cells. Vinorelbine was then entrapped into the liposomal vesicles as an antitumor drug. This modification results in multifunctional targeting drugs-loaded liposomes that could enhance the transport drugs across the BBB, increase the intracellular uptake, inhibit all glioma cells and induce apoptosis via activating related apoptotic proteins. Moreover, the in vivo results showed that multifunctional liposomes could significantly accumulate into brain tumor location, specific to tumor sites, and robust overall antitumor efficacy.

Another group reported the use of cyclic RGD (c(RGDyK)) and p-hydroxybenzoic acid (pHA) for multifunctional glioma-targeted drug delivery [103]. The mentioned peptides were developed to target integrin αvβ3 overexpressed on the blood-brain tumor barrier (BBTB), glioma cells, and dopamine receptors on the BBB, respectively. In this present work of Belhadj’s group, pHA and c(RGDyK) were both modified on the surface of PEGylated liposomes, while doxorubicin (DOX) was chosen as the chemotherapeutic agent for glioma treatment. In vitro tests demonstrated that c(RGDyK)/pHA modification on the drug-loaded liposomes plays a major role in the transport of the multi-targeting liposomes across the BBB and BBTB models and pHA modification enhanced the transport of liposomes across the BBB, as proven by the results of inhibition assay, while the existence of c(RGDyK) on the surface of liposomes enhanced the transcytosis efficiency of the multifunctional targeting liposomes across the BBTB. Besides, an in vivo fluorescence imaging study showed that c(RGDyK)/pHA-LS in the glioma site was much more than other groups, which denoted that c(RGDyK)/pHA-LS had a strong glioma targeting effect. Moreover, survival study enhanced that antiglioma efficacy of c(RGDyK)/pHA-LS could be probably owing to the c(RGDyK)/pHA functionalization, which could facilitate the accumulation of more liposomes in the tumor site, and further drive DOX-loaded liposomes internalization into glioma cells.

Similarly, Zing et al. conjugated the cyclic derivative (cA7R) on the surface of DOX-loaded PEGylated liposomes [104]. The system exhibited excellent antitumor, anti-angiogenesis, and anti-vasculogenic mimicry effects, resulting in improved therapeutic efficacy in U87 xenograft nude mice as compared to DOX solution, DOX-loaded non-functionalized liposomes, or DOX-loaded liposomes that were functionalized with LA7R.

In addition, Fu and fellow workers prepared a functionalized liposome with TAT and cleavable PEG via a redox-responsive disulfide linker for the delivery of Paclitaxel (PTX-C-TAT-LP). These in vitro and in vivo results confirmed a superior delivery efficiency of PTX-C-TAT-LP for enhancing tumor distribution [105].

From the above discussion, liposomal surfaces have been modified while using a variety of approaches to generate PEGylated, ligand targeted, and multifunctional liposomes. Based on the recent advances in liposomal surface modifications, it is worth acknowledging the various liposome-based carrier systems that have been used or hold the promise of being used in the future to deliver therapeutic substances to different organs and tissues in the body.

## 4. Current Applications in Liposome Surface Modification for Drug Delivery

Liposomes are part of the most reputed carriers of various molecular species, ranging from small and simple to large and complex molecules. As Nkanga et al. mention, the spherical sacs of phospholipid molecules are artificial lipid-based bilayered vesicles that have been subject to extensive evolution [106]. Notably, liposomes have evolved in terms of composition, manufacturing, and application to be used in both basic and applied life sciences. One of the evolutions is the modification of soy lecithin liposome. For instance, Tiwari et al. conducted a study to formulate and evaluate a new combination of 5 Flourouracil and Tretinoin for topical administration [107]. The study indicated that the development and optimization of liposomes containing 5 Fluorouracil and Tretinoin was effective in the control of skin warts with stability and efficient drug release. Liposomes have also been adapted for oral drug delivery due to their ability to mimic natural cell membranes. According to He et al., modifying liposomes improves the bioavailability of liposomes and their opportunities to be taken up by intestinal epithelia [108]. In this sense, liposomes are prone to degradation due to gastric acids, bile salts, and pancreatic lipases, which results in leakage of the payloads. Besides, a study by Hirose et al. to evaluate the effect of soy lecithin on fatigue and menopausal symptoms in middle-aged women found that lipid replacement therapy could repair the damaged cell membranes to alleviate fatigue [109]. The results indicate that a high-dose of soy lecithin liposome, 1200 mg per day, increased vigor, lowered diastolic blood pressure, and reduced cardio-ankle vascular index in middle-aged women. The modifications that were made to soy lecithin liposome have advanced healthcare delivery through drug delivery.

It is notable that surface modification of liposomes is determined by the specific application of the liposomes (Table 2). Therefore, this section shall discuss how liposome surface modification is applied in cancer treatment, brain targeting, and vaccinology.

### 4.1. Cancer Treatment

The application of liposomes in drug delivery is divided into two main sections: cancer therapy and applications in other fields, such as vaccinology and gene therapy [131,132,133]. Therefore, cancer therapy applications are very important in exploring how liposomes are modified to create anticancer formulations. Kobayashi, Tsukagoshi, and Sakurai first demonstrated the potential of liposomes in the management of cancer, as cited in Roberto et al. [50,134,135]. In the study, Kobayashi et al. showed that cytosine arabinoside could be encapsulated in liposomes and delivered effectively in L1210 leukemia cells, thus increasing the survival of the affected mice [134]. Following this revelation, L1210 leukemia in mice became the model for evaluating the efficiency of liposome drug delivery systems in cancer therapy [50].

A major challenge that is often encountered in cancer treatment is the side effects of anticancer agents and low drug accumulation at the tumor site [136,137,138]. Additionally, the delivery of anticancer drugs is interrupted by phagocytosis and other defense mechanisms of the body [21,139,140]. Liposomes that are used as drug delivery vehicles are coated with polyethylene glycol (PEG) to resolve these problems. The use of PEGylated liposomes as a passive targeting strategy in cancer therapy can be enhanced by creating liposomes of very small sizes (40–200 nm). Moreover, higher extravasation is seen in liposomes of such size range, as they are able to traverse the membranes of cancer cells more efficiently and promote drug retention [91].

The effectiveness of PEGylated liposomes for drug delivery in tumors was evaluated while using doxorubicin. Human models confirmed that the anticancer drug exhibited longer circulation times when it was entrapped in stealth liposomes compared to conventional liposomes [50] Stealth liposomes were further tested during the first clinical trial of entrapped doxorubicin in HIV patients with Kaposi’s sarcoma. It was noted that PEGylated liposomes enabled the drug to stay longer in circulation and accumulate in sites where vascularization was high (inflammatory and tumor sites) [141]. Despite this positive observation, it appeared that the therapeutic index (TI) and efficacy of doxorubicin could be enhanced by making the liposomes more selective in targeting the diseased cells [141].

**Figure 4:**
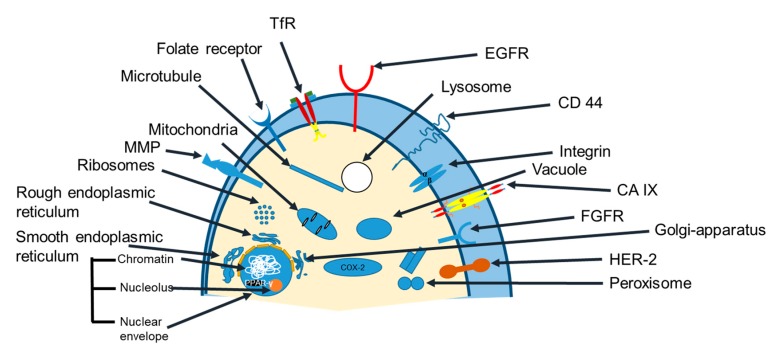
Receptors (intracellular and extracellular) that can be targeted using ligand-targeted liposomes for the delivery of anticancer drugs. Targets could also be specific organelles (mitochondria and lysosomes) [21].

The need for selective targeting of tumor cells has led to the development of liposomes with functionalized surfaces. For example, targeting ligands (peptides, antibodies, proteins) are coupled to the liposome surface to enhance the selective attachment of the liposomes on cells bearing certain receptors on their surfaces [50]. In cancer therapy, a variety of extracellular receptors (see Figure 4) are targeted with liposomes carrying anticancer agents, such as Vincristine, Paclitaxel, and Doxorubicin. Depending on the targeting ligand to be used, it can be attached to the end of PEG molecules or directly on the lipid bilayer of the liposome. During the design and development of targeted liposomes for the delivery of anticancer drugs, it is important to optimize the density of the ligand by applying the appropriate surface modification techniques. The advantages of using targeted liposomes in cancer treatment include better drug internalization by cancer cells and reduced nonspecific drug distribution [21].

As can be seen in Figure 4, folate receptors (FRs) are potential targets of ligand-targeted liposomes. Their overexpression on the surfaces of cancer cells in breast and lung cancers have been reported by Low, Henne and Doorneweerd [142]. While using stealth liposomes, Low et al. conjugated folate to PEG moieties and incorporated doxorubicin into the liposomal formulation. The researchers noted higher cytotoxicity of the FR-targeted liposomes than liposomes containing doxorubicin but lacking the targeted functionalization. Ligands that are used to modify the surfaces of liposomes for cancer therapy can also be targeted at receptors that are over-expressed in the cytoplasm of cancer cells. Alternatively, the key organelles within the cytoplasm that play important roles in the development of cancer can be effectively targeted (see Figure 4). For example, Biswas et al. (2011) and Biswas et al. (2012) incorporated paclitaxel in liposomes that had been modified while using either triphenylphosphonium moiety or rhodamine 123 for mitochondrial targeting [82,143]. It was observed that the use of these ligands resulted in higher specificity and cytotoxicity in the affected mitochondria than liposomes without ligands.

In addition to developing targeted liposomes, as outlined above, researchers have explored the potential of multifunctional liposomes in cancer therapy. Such liposomes carry more than one ligand in order to improve their drug delivery and targeting efficacy. In a study that tested the usefulness of multifunctional liposomes, Zhang et al. prepared liposomes by attaching peptide DA7R (to target VEGFR2) and peptide T7 (to target TfR) to the surfaces of the liposomes and incorporating two anticancer agents (vincristine and doxorubicin) [99]. The dual targeted strategy against brain tumor demonstrated improved delivery of the drugs and better treatment outcomes.

### 4.2. Brain Targeting

Neurodegenerative diseases are a major health burden, especially in the developed world, where they are among the four leading causes of death [144]. Alzheimer’s disease, prion disease, and Lewy body dementia are among the most prevalent neurodegenerative diseases. Despite the great need to develop medical interventions, the protective barriers that surround the central nervous system have hampered the efficacy of various treatments. Barriers such as BBB make it very difficult for neurotherapeutics to reach the neurons. However, liposome technology has provided a viable mechanism of delivering new therapeutic drugs to the brain.

**Figure 5:**
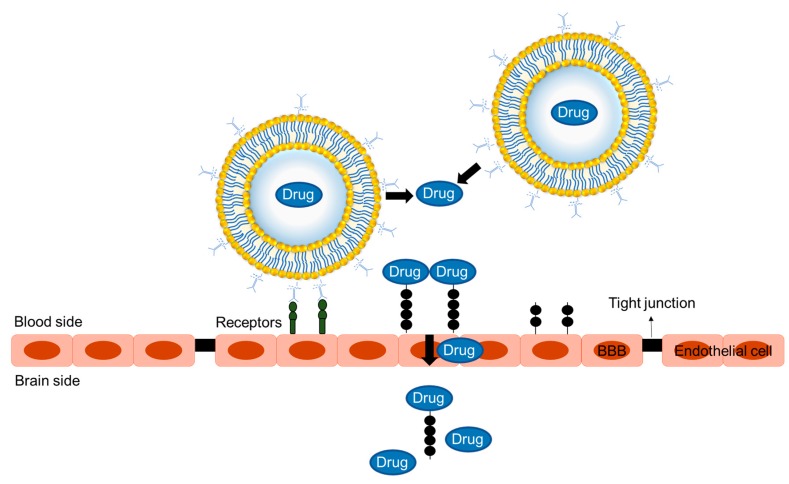
The mechanism of drug delivery across the BBB using ligand-targeted immunoliposomes.

Some studies show that liposomes whose surfaces have been modified with appropriate ligands are able to cross the BBB and release their drug content or genetic material into the affected brain without damaging the brain. The ligand on the liposomes binds to the specific receptor on the BBB (see Figure 5). This is followed by endocytosis through endocytotic vesicles that facilitate the release of the drug into the intracellular space, followed by transcytosis [145].

Most ligands that are used to modify the surfaces of liposomes for the delivery of neurotherapeutics target lipoprotein and transferrin receptors. For example, targeted liposomes carrying an antibody called OX26 specifically binds to transferrin receptor and can be used to cross the BBB to deliver important drugs, hormones, or growth factors [145]. In one of the studies, Monsalve et al. developed PEGylated liposomes and further conjugated the PEG molecules to chitosan (CS) before attaching the ligand (OX26). Another sample of liposomes lacking the targeting ligand was also formulated. It was observed that the PEG-CS-OX26 liposomes exhibited selective binding to transferrin receptors and were able to traverse the BBB [146].

### 4.3. Vaccinology

Liposomes have been studied in terms of their potential applications in the delivery of important vaccines for the prevention of various diseases, including TB, malaria, and hepatitis. Their application in this field is based on the modification of their cargo as well as lipid composition. For example, liposomes for vaccinology applications can be modified by incorporating antigens in the form of peptides, lipids, or nucleic acids [147]. Alternatively, molecular patterns that are associated with the pathogen can be used to enhance the adjuvant properties for the modulation of inflammatory responses at the sites where T lymphocytes are primed [50]. It is worth noting that research into the usefulness of surface-modified liposomes in vaccinology has led to the development of clinically viable liposome formulations and liposome-based vaccines against TB, HIV, malaria, and dengue fever. Additionally, several liposome vaccines, that is, Inflexal, Epaxal, and Cervarix, are available in the market for applications against influenza virus, hepatitis A virus, and human papillomavirus (HPV), respectively [50]. These commercially available vaccines are produced by reconstituting viral envelope and supplementing the formulation with phosphatidylcholine. Therefore, these vaccines are generally classified as virosomes. The main advantage of using the vaccines is that they have the capacity to fuse with the membranes of the endosomes or the targeted cells for efficient delivery of their cargo in the cytosol [106].

Lipids are very important molecules during the production of liposome vaccines, as they function as second messengers during the regulation of molecular pathways that control the maturation of phagosomes and the subsequent antimicrobial responses [50]. The ability of lipids to act as second messengers hence makes them valuable molecules for liposome modification to produce targeted liposomes. These targeted liposomes have the ability to selectively attach to infected cells and to deliver the specific second messenger for prompt activation of molecular pathways against specific pathogens. For example, liposomes that are composed of dimethyldioctadecylammonium (a positively charged lipid) and trehalose 6,6′-dibehenate (a glycolipid) in the mass ratio 5:1 have been shown to be efficient delivery systems of Ag85B-ESAT-6 (a TB vaccine). Additionally, the immune response following the administration of the liposome TB vaccine is prolonged and without toxic effects [106].

Apart from lipids, other liposome function modifiers, such as pathogen-associated molecular patterns (PAMPs), can be used to enhance the adjuvant properties of the liposomes. PAMPs added to the liposome structure facilitate the development of liposomes that play a central role in the activation and modulation of antigen-presenting cells (APCs) [50]. The modulatory function of the functionalized liposomes includes the modulation of the cytokine profile. This cascade then induces T helper cell differentiation [50]. For example, an immune response that was Th1-oriented was induced in mice following their immunization with liposome vaccines carrying oligodeoxynucleotide and ovalbumin. These PAMPs carried CpG motifs that acted as ligands for Toll-like receptors (TLR-9). The resultant immune response was accompanied by the increased production of IgG2a and IFN-γ [148]. In a similar study, a different ligand (Pam3CSK4) was conjugated with oligodeoxynucleotide and ovalbumin to target TLR-2. This time, the immunized mice showed enhanced Th2-immune response as confirmed by the heavy production of IgG1 [50]. In a nutshell, liposomes can be modified to accommodate antigens within the bilayer or on their surfaces, thus triggering different responses by the immune system.

## 5. Conclusions

Over the past one or two decades, research into the use of liposomes as efficient drug delivery vehicles has generated encouraging outcomes in the management and treatment of different diseases. Following the discovery of liposomes, the research fraternity has focused on designing and applying different surface engineering techniques. The application of the surface modification strategies has given rise to advanced liposomal formulations that are able to overcome the limitations of conventional liposomes. It is notable that a good number of credible studies on the surface modification of liposomes have been successful. However, there is evidence of a gap between the discovery of liposome surface modification and their application in human models. Future studies should aim at demonstrating the success story through clinical trials that are aimed at resolving various public health challenges while using liposome-based drug delivery systems. Soy lecithin-derived liposomal delivery systems are particularly promising as a lot has been accomplished in terms of their application in major illnesses, such as TB and malaria. However, it is also evident that the transition from mice models to human clinical trials has been slow. Going forward, researchers should conduct more clinical trials using soy lecithin-derived liposomal delivery systems.

## Figures and Tables

**Table 1:** Comparison of particle size and casein hydrolysate (CH) entrapment efficiencies of the tested liposome formulations as reported by Yokota et al. [39].

Formulated Liposome	Efficiency of Casein Hydrolysate Encapsulation (%)	Average Vesicle Size (μm)
Non-cryoprotected CH-loaded S40 liposomes	46.0 ± 1.0	0.53 ± 0.02
Non-cryoprotected CH-loaded S100-H liposomes	43.4 ± 1.75	5.00 ± 1.21
CH-loaded S40 liposomes cryoprotected using sucrose	30.8 ± 1.22	2.27 ± 0. 31
CH-loaded S40 liposomes cryoprotected using trehalose	37.1 ± 0.80	2.15 ± 0.23

**Table 2:** Liposomal commercialized products in USA market. (Data from US FDA approved).

Products (Approval Year)	Administration	Drug	Particle Type	Lipid Composition	Indication	Ref
Doxil^®^ (1995)	Intravenous	Doxorubicin	PEGylated liposome	HSPC, cholesterol and DSPE-PEG_2000_	Kaposi’s sarcoma, ovarian and breast cancer	[110,111,112]
Lipo-dox^®^ (1995)	Intravenous	Doxorubicin	PEGylated liposome	DSPC, cholesterol and DSPE-PEG_2000_	Kaposi’s sarcoma, ovarian and breast cancer	[112]
DaunoXome^®^ (1995)	Intravenous	Daunorubicin	Non-PEGylated liposome	DSPC and cholesterol	Blood cancer	[113,114]
Myocet^®^ (1996)	Intravenous	Doxorubicin	Non-PEGylated liposome	EPC and cholesterol	Metastatic breast cancer	[115,116]
Ambisome^®^ (1997)	Intravenous	Amphotericin B	Non-PEGylated liposome	HSPC, DSPG and cholesterol	Sever fungal infections	[117]
Depocyt^®^ (1999)	Spinal	Cytarabine	Non-PEGylated liposome	DOPC, DPPG, cholesterol and triolein	Neoplastic meningitis and lymphomatous meningitis	[118]
Visudyne^®^ (2000)	Intravenous	Verteporfin	Non-PEGylated liposome	EPG and DMPC	Age-related molecular degeneration	[119,120,121]
DepoDur^®^ (2004)	Epidural	Morphine sulfate	Non-PEGylated liposome	DOPC, DPPG, Cholesterol and Triolein	Pain management	[122,123,124]
Marqibo^®^ (2012)	Intravenous	Vincristine	Non-PEGylated liposome	SM:Cholesterol (60:40 molar ratio)	Acute lymphoblastic leukaemia	[125,126,127]
Onivyde^®^ (2015)	Intravenous	Irinotecan	PEGylated liposome	DSPC:MPEG-_2000_:DSPE (3:2:0.015 molar ratio)	Combination therapy with fluorouracil and leucovorin in metastatic adenocarcinoma of the pancreas	[128,129,130]
